# First insights in the variability of *Borrelia recurrentis* genomes

**DOI:** 10.1371/journal.pntd.0005865

**Published:** 2017-09-13

**Authors:** Durdica Marosevic, Gabriele Margos, Reinhard Wallich, Andreas Wieser, Andreas Sing, Volker Fingerle

**Affiliations:** 1 German National Reference Centre for Borrelia, Bavarian Health and Food Safety Authority (LGL), Oberschleißheim, Germany; 2 European Programme for Public Health Microbiology Training, European Centre of Disease Prevention and Control (ECDC), Stockholm, Sweden; 3 Medical Faculty, University Hospital Heidelberg, Germany; 4 Department of Bacteriology, Max von Pettenkofer Institute (LMU), Munich, Germany; 5 Division of Infectious Diseases and Tropical Medicine, Medical Center of the University of Munich (LMU), Munich, Germany; 6 German Center for Infection Research (DZIF), Partner Site Munich, Munich, Germany; Medical College of Wisconsin, UNITED STATES

## Abstract

**Background:**

*Borrelia recurrentis* is the causative agent of louse-borne relapsing fever, endemic to the Horn of Africa. New attention was raised in Europe, with the highest number of cases (n = 45) reported among migrants in 2015 in Germany and sporadically from other European countries. So far only one genome was sequenced, hindering the development of specific molecular diagnostic and typing tools. Here we report on modified culture conditions for *B*. *recurrentis* and the intraspecies genome variability of six isolates isolated and cultured in different years in order to explore the possibility to identify new targets for typing and examine the molecular epidemiology of the pathogen.

**Methodology/Principal findings:**

Two historical isolates from Ethiopia and four isolates from migrants from Somalia (n = 3) and Ethiopia (n = 1) obtained in 2015 were cultured in MPK-medium supplemented with 50% foetal calf serum. Whole DNA was sequenced using Illumina MiSeq technology and analysed using the CLC Genomics Workbench and SPAdes *de novo* assembler. Compared to the reference *B*. *recurrentis* A1 29–38 SNPs were identified in the genome distributed on the chromosome and plasmids. In addition to that, plasmids of differing length, compared to the available reference genome were identified.

**Conclusions/Significance:**

The observed low genetic variability of *B*. *recurrentis* isolates is possibly due to the adaptation to a very conserved vector-host (louse-human) cycle, or influenced by the fastidious nature of the pathogen and their resistance to *in vitro* growth. Nevertheless, isolates obtained in 2015 were bearing the same chromosomal SNPs and could be distinguished from the historical isolates by means of whole genome sequencing, but not hitherto used typing methods. This is the first study examining the molecular epidemiology of *B*. *recurrentis* and provides the necessary background for the development of better diagnostic tools.

## Author summary

Louse-borne relapsing fever, as the name suggests, is the only relapsing fever transmitted by lice, and caused by the spirochaete *Borrelia recurrentis*. Today it is endemic to the Horn of Africa, but due to the cosmopolitan nature of the vector, the pathogen still bears epidemic potential to spread globally among vulnerable populations. The most recent account of that has been observed among migrants arriving to Europe in 2015. Up to date, only one strain was sequenced, thus hampering the development of species-specific typing tools. We employed state-of-the-art high-throughput sequencing to six *B*. *recurrentis* isolates obtained at different time-points and currently available in culture. Our aim was to address the question of genome variability of this pathogen at the highest resolution and provide information necessary for the development of specific typing tools. *B*. *recurrentis* has highly conserved genomes, differing in 29–38 SNPs compared to the reference genome *B*. *recurrentis* A1, all identified outside the loci currently developed and used for relapsing fever *Borrelia* typing. Therefore, applying these typing methods would render them indistinguishable, while at the SNP level we found a distinction between isolates obtained in 2015 from migrants and the two historical isolates. Our data provide first insights in the genome variability and baseline information necessary for further studies of the molecular epidemiology of the pathogen and for the development of improved diagnostic tools.

## Introduction

*Borrelia recurrentis* is the causative agent of human louse-borne relapsing fever (LBRF). It is the only relapsing fever *Borrelia* species transmitted by the body louse *Pediculus humanus* [1], and–apart from humans—has no known animal reservoir hosts, nor other vectors [2]. There are 22 different currently known tick-borne relapsing fever (TBRF) *Borrelia* species that are transmitted by different soft and hard ticks, and that have different natural animal reservoir hosts [3, 4]. The classification and diagnosis of TBRF historically relied often on the vector identification and geographical region [5]. These host and vector specificities of RF borreliae have an impact on human infections, their public health importance and their geographic distribution [3, 5, 6].

Due to the cosmopolitan nature of the human body louse, *B*. *recurrentis* was once present worldwide and affected millions of people, causing major outbreaks during times of war and crisis [2, 6]. Improvement of hygiene and living conditions has led to a decrease of body lice infestations in the industrialized world, and the pathogen is nowadays endemic to the Horn of Africa [7, 8], where it is still a major public health concern. It is the seventh most common cause (up to 27%) of hospital admission and the fifth most frequent cause of death in the highlands of Ethiopia [9–11]. Limited data are available on the prevalence of the disease in neighboring countries such as Sudan, the Republic of South Sudan or Somalia [7, 12, 13]. LBRF and TBRF differ not only in the mode of transmission, but also in the severity and the outcome of the disease. LBRF is characterized by a three to ten day incubation period, one to five relapses of fever that typically last three to five days, with afebrile intervals that lengthen as the disease progresses. It has a poorer outcome compared to TBRF [3]. If left untreated mortality of LBRF can reach up to 40%, while for TBRF it is below 5%. Even if treated, LBRF mortality can be as high as 10% [3], mostly due to a severe reaction caused by pro-inflammatory cytokines called Jarisch-Herxheimer (JH) reaction [14]. Whether the severity of the relapsing fevers and development of JH reaction is somehow influenced by the genetic and antigenic make-up of *B*. *recurrentis* is not clear and to the best of our knowledge was not studied so far.

Molecular typing tools such as PCR of protein coding genes, intergenic spacer typing, or the sequencing of housekeeping genes developed for TBRF *Borrelia* species [15] showed limited success when applied to *B*. *recurrentis*. It proved especially difficult to differentiate the two closely related spirochaetes *B*. *recurrentis* and *B*. *duttonii* [16]. This high similarity casted doubt on the species separation, and it was suggested that they are ecotypes of the same species, recently adapted to, and evolving in, different vectors [3, 15, 17]. The distinction of these closely related relapsing fever spirochaetes is important due to the fact that antibiotic treatment of LBRF leads more often to JH reactions and requires intensive care [14].

Up to now, only one whole genome sequence is available for *B*. *recurrentis* [17]. The *B*. *recurrentis* strain A1 contains eight linear genome fragments, the longest with 930 kb was designated as the chromosome and the remaining seven as plasmids (pl124, pl53, pl37, pl35, pl33, pl23, pl6) named according to the identified length in kb. Although this provided first insights into the genetic composition, the availability of only one genome does not provide insights into the population variation and, thus, hampered the development of more specific typing tools. It was, for example, not possible to address questions such as genome variability of the species and the potentially differing virulence or the molecular epidemiology of different isolates. Further complications for genome comparison and development of typing and/or diagnostic tools arise from the fact that *B*. *recurrentis* are relatively rare and difficult to culture in axenic medium or animal models [18]. Until the 1990s the species was deemed non-cultivable due to their fastidious and slow growing nature [19, 20], and even nowadays success in cultivation is limited [3].

*B*. *recurrentis* today still bears a considerable potential to occur and spread among vulnerable populations that are exposed to unfavorable conditions, with limited access to sanitation and personal hygiene facilities and in overcrowded circumstances where louse infestations are common [21–23]. The most recent example of *B*. *recurrentis* infections have been observed in Europe during 2015 [8, 24–29]. All cases were imported, and associated with newly arrived migrants, with a possible autochthonous transmission in Italy [24]. Interestingly, the majority of cases reported in Bavaria, Germany (92%) were among people from Somalia, while 3% were from Ethiopia and 5% from Eritrea [30]. Similar observations were made in other European countries, the majority of reported cases were among Somalian refugees, while in the Netherlands and Switzerland reported cases were only from Eritrea.

Here, we report on conditions that enabled us to culture a number of isolates from refugees from Somalia, Eritrea and Ethiopia. This provided the opportunity to explore the intraspecies genome variability of six *B*. *recurrentis* isolates. To the best of our knowledge, this is the first study examining the genomic variability of *B*. *recurrentis* with state-of-the-art next generation sequencing techniques and it provides valuable information necessary for the development of new diagnostic and typing tools and a better understanding of the molecular epidemiology of this pathogen.

## Materials and methods

### Samples included in the study

Two isolates were historical, isolated from subjects infected in Ethiopia in 1985 [31] and 2004, and four isolates were isolated in 2015 from subjects arriving from Somalia (n = 3) and Ethiopia (n = 1). Due to long migration routes that were only vaguely described the exact place of infection is unclear and can only be speculated for the four isolates from 2015 (Table 1).

**Table 1:** Isolates examined in this study.

Isolate	Year of isolation	Country*	Reference
A17	1985	Ethiopia	[31]
PBeK	2004	Ethiopia	This study
PAbN	2015	Ethiopia	This study
PAbJ	2015	Somalia	This study
PMaC	2015	Somalia	This study
PUfA	2015	Somalia	This study

*Country, for the two historical isolates means country of infection. For the four isolates isolated in 2015, country is the country of origin of the patients, due to long and strenuous travel the exact place of infection is not clear.

### Culture conditions

Blood samples were sent to the Bavarian Health and Food Safety Authority for diagnostic purposes. 20 μl of the samples were placed on a microscope slide, covered with a cover slip and viewed using dark field microscopy Zeiss Axioscope Microscope, objective 40X, ocular 10X. To aid diagnostics, 500 μl of blood sample were placed in to Modified-Kelly-Pettenkofer (MKP) culture medium [32] that was additionally supplemented with 50% foetal calf serum (FCS). Cultures were kept in screw cap glass tubes (7 ml volume) at 33°C. Cultures were examined on a regular basis (2 x per week) to check for growth of bacteria. Actively growing bacterial cultures were subcultured or diluted using MKP-50% FCS medium. Aliquots of well growing cultures were frozen according to standard methods with 15% Glycerin [32].

### DNA extraction, library preparation, sequencing

Total DNA was isolated from 35 ml of culture with a cell density of appr. 5X10^6^ - 1X10^7^ using the Maxwell 16 LEV Blood DNA Kit according to the manufacturer's instruction on the Maxwell Instrument (Promega, Germany).

DNA libraries for whole genome sequencing were constructed using the Nextera XT DNA Library Preparation Kit (Illumina, San Diego, USA). Samples were sequenced using Illumina MiSeq technology and a V2 Reagent Kit (Illumina) to produce 2x250 bp paired-end reads according to the manufacturer's instructions.

### Sequence data analyses

Analysis was performed using the CLC Genomics Workbench 9.0.1 software. Briefly, reads were trimmed using quality scores: the limit was set to 0.05, and allowing for 2 ambiguous nucleotides, with a minimum length of 50. Contaminations with human DNA were removed by mapping all reads to a human reference genome (GCA_000001405.23), with the following mapping options: match score 1, mismatch cost 2, insertion and deletion cost 3, similarity fraction 0.8 and length fraction 0.5. The remaining reads were both, *de novo* assembled and mapped to the reference genome *B*. *recurrentis* A1 (GCA_000019705.1).

Read mapping was performed using the integrated CLC mapper, with the same conditions as mentioned above for mapping to the human genome. Variant calling was performed with the CLC tool “Basic Variant Detection”, with ploidy set to 1, ignoring positions with coverage above 100.000, broken pairs and non-specific matches. The minimum read length was set to 20, minimum coverage 10 and minimum frequency of 90%. Quality filters to the neighboring 5 nucleotides included a minimum central quality of 20, and neighborhood quality 15.

For *de novo* assembly two different software were used: the *de novo* assembler provided in CLC and the SPAdes assembler version 3.9.0 [33]. In CLC, the same mismatch, insertion and deletion costs as for mapping were applied, with an automatic bubble (50) and k-mer (20) size. Alignment mode was set to local and match mode to random, minimum contig length was 500 bp. *De novo* assembly in SPAdes was performed using the following settings: the “careful mode” was chosen in order to reduce mismatches and short indels, k-mer sizes included 21, 33, 55, 77, 99, 127. *De novo* assembled contigs were aligned to the reference genome A1 using the CLC Microbial finishing module, MAUVE [34]; and QUAST [35], and visualised using BRIG [36]. MEGA software version 7.02 [37] was used for phylogenetic tree construction using the identified SNP positions with the maximum likelihood method, and genetic distance matrixes corrected using the Tamura-Nei substitution model [38]. The topology of the trees obtained was assessed by bootstrapping, with 1000 replications. A large scale synteny analysis of *de novo* assembled contigs and the reference genome *B*. *recurrentis* A1 was performed using MUMmer 3.0 [39].

### PCR primers and conditions

Primers specific for the 5’ end of the plasmid pl6 and 3’ end of the pl165 were designed (Table 2). PCR was performed with HotStarTaq PCR from QIAGEN with the following conditions: 15 min at 95°C followed by 30 cycles 1 min at 94°C, 1 min at 50°C, 1 min extension at 72°C with a final extension step at 72°C for 10 min.

**Table 2:** Primers used for confirmation of plasmids in *B*. *recurrentis*.

pl6_F2	5´-TGGCACCATTATCTTTCCAGTTG-3´
pl6_R2	5´-CGGGAGTGTTTGGGGTTACA-3´
pl6_F3	5´-ATCCCAATTGGATAGGGGGA-3´
pl6_R3	5´-TTGCTCGCTCAAAGCTTCTT-3´
pl165_R1	5´-CGTGATACTGTTGTTTGGG-3´
pl165_F3	5´-TTACATCGCACCAAAAGGC-3´

#### Sequence deposition

All sequences have been deposited at the GenBank SRA under accession number: SRP101704.

## Results

During 2015, blood samples from 38 patients diagnosed with LBRF were available at the Bavarian Health and Food Safety Authority/National Reference Centre for *Borrelia*, thereof 21 could be adapted to the modified culture conditions described in this study. Initially, culture was attempted in BSK, MPK and MPK supplemented with 50% FCS. This was the medium of choice in our laboratory for tick-borne relapsing *Borrelia*, and proved to be the most successful for *B*. *recurrentis*. No growth was observed in BSK, nor MPK medium. *B*. *recurrentis* strains from 9 samples had grown to acceptable densities (10^6^−10^7^/ml) and subjected to DNA extraction. Four isolates from 2015 yielded DNA of sufficient quantity and quality for sequencing. In addition, the two historical isolates that were well adapted and brought to culture in 1985 (A17) and 2004 (PBek) were included. The isolate A17 had been recovered from a 15 year old male in Ethiopia and the isolate PBek had been isolated at the German National Reference Centre for Borrelia from a traveler returning from Ethiopia, and was suspected to belong to the *B*. *recurrentis* species. We were able to confirm this species designation in this study.

**Fig 1:**
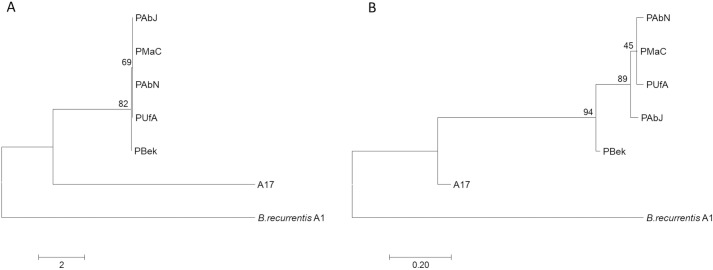
A phylogenetic tree based on 23 SNP positions identified on the main chromosome. Evolutionary analysis was performed using MEGA7. B Phylogenetic tree based on 47 SNP positions identified on the whole genome of *Borrelia recurrentis*. The evolutionary history was inferred by using the Maximum Likelihood method based on the Tamura-Nei model.

Total reads per sample ranged from 736,000–2,203,405. Read mapping to the A1 reference genome available in GenBank resulted in an average coverage between 52 and 1094 for all plasmids and the main chromosome, and >99% of the genome had a coverage of ≥10x (Table 3). In isolates obtained from migrants, a total of 17 single nucleotide polymorphisms (SNPs), identical in all four isolates, compared to the main chromosome of A1 were identified. The two historical isolates A17 and PBek had 12 and 16 SNPs, respectively (with a min. coverage of 10 and a min. frequency 90%) (see S1 Table and Fig 1A). Six SNPs were common to all isolates, thereof two in non-coding regions, one was a silent mutation, and three nonsynonymous mutations in a serine protease, a permease and in the apparent leading methionine in an UDP-N-acetylmuramoyl-tripeptide—D-alanyl-D-alanine ligase. In addition to the common 6 SNPs, the isolates recovered from migrants in 2015 and the historical isolate PBek shared 10 more SNPs compared to the chromosome of the reference strain A1. All were located in coding regions, three synonymous changes in UvrC, chemotaxis protein CheW and a trigger factor, while seven were nonsynonymous and would lead to amino acid changes in a transcript cleavage factor, dipeptide/oligopeptide/nickel ABC transporter ATP-binding protein, GyrB, 30S ribosomal protein S5, chemotaxis protein, tRNA methyltransferase TrmD, transcription termination/antitermination protein NusA. The isolates from 2015 had one more SNP causing an amino acid change in the acriflavin resistance protein. The isolate A17 differed the most compared to the other six sequenced genomes. It contained one SNP in a noncoding region, one change in the 23S rRNA, an additional SNP in the aforementioned serine protease and 3 SNPs causing amino acid changes in the genes coding for the flagellar motor switch protein FliG, peptidase M16, peptide ABC transporter permease.

**Table 3:** Number of sequenced reads per sample mapped to the reference genome *B*. *recurrentis* A1.

Name	# reads after quality trimming	# reads mapped to human DNA	# reads mapped to reference A1	Average coverage*	% of genome with ≥10 coverage	% of unmapped reads	Max. contig length from *de novo* assembled unmapped reads
A17	1,295,220	28,515	1,232,558	171–688	99.91	2.62	19,509
PBek	1,912,724	51,602	1,812,678	252–1094	99.83	2.53	17,244
PAbJ	736,413	49,571	665,694	105–339	99.63	3.06	12,726
PAbN	1,329,463	870,338	443,357	52–198	99.91	3.48	17,242
PMaC	899,415	109,432	766,990	109–392	99.91	2.91	12,728
PUfA	2,203,405	1,571,601	608,816	75–243	99.63	3.64	17,242

*Average coverage was calculated for chromosome and every plasmid separately

**Fig 2:**
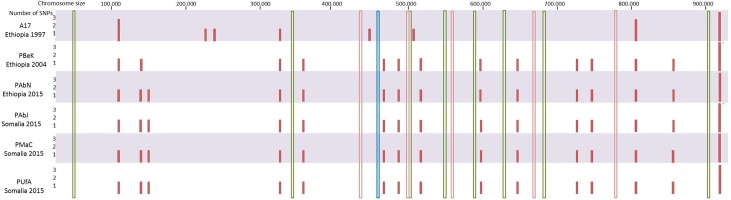
Location of the identified SNPs on the main chromosome (full pink bars) in comparison to the loci used for typing of Borrelia species IGS (blue), MST (pink) and MLST (green).

Performing SNP calling with less stringency (min. coverage 10 and min frequency 75% or 50%), identified two additional SNPs. At 75% a deletion in a poly-A region was detected in all refugee isolates and in the PBek historical isolate and at 50% in the PBek isolate one more SNP was called in a coding region. Both SNPs would lead to a frameshift mutation. Notably, all identified SNPs were outside of the 14 loci developed and used for *Borrelia* typing [16, 40, 41]. This includes the 8 housekeeping genes used for the MLST typing of other *Borrelia* species [41], the 5 loci used in MST [16] and the intergenic spacer typing (IGS) [40] method developed for typing of *B*. *recurrentis* and *B*. *duttonii* (Fig 2). Mapping the reads to *B*. *duttonii* (GCF_000019685.1), identified 185–260 single nucleotide differences over the main chromosome, highlighting potential loci for the development of specific molecular diagnostic tools.

All seven plasmids present in the A1 reference genome were identified via read mapping in the newly sequenced isolates. Between 17 and 21 SNPs on five of the seven plasmids (pl124, pl23, pl33, pl35 and pl53) were identified in all samples compared to the reference *B*. *recurrentis* A1 genome (Table 1 in supplementary data). Majority of SNPs (n = 14) were present at the furthest ends (250 bp) of the linear plasmids. In two of the seven plasmids no SNPs were identified (pl37 and pl6). The plasmid SNPs from isolates recovered in 2015 were not identical as was the case for the chromosomal SNPs, nevertheless, those isolates were more related than the historical isolates as illustrated by the SNP phylogenetic tree (Fig 1B).

Employing the *de novo* assembly approach, it was possible to assemble draft genomes of the six *B*. *recurrentis* isolates in 25–54 contigs with SPAdes and 41–70 contigs with the *de novo* assembler of CLC (Tables 4 and 5). At least 98.96% of the contigs assembled had a coverage ≥ 10x, and the draft genome of the isolate PBek had 99% of the contigs covered ≥ 20-fold (Tables 4 and 5). The main chromosome could be reconstructed in 1–2 contigs, and no large gene rearrangements, deletions, insertions, translocations, could be observed (see supplementary data S1 Data).

**Table 4:** Statistics of *de novo* assembled contigs with CLC *de novo* assembler.

Name	# denovo contigs (min length 500)	Max length	N50	Average coverage*	% of contigs with ≥10 coverage	# misassemblies	Total length of misassembled contigs
A17	48	226,028	117,719	6–2,651	99.93	1	10,757
PBek	56	226,028	117,717	5–4,113	99.85^#^	1	10,840
PAbJ	56	255,534	93,247	5–761	99.85	1	10,834
PAbN	50	226,024	145,563	24–853	99.68	1	4,813
PMaC	41	226,026	145,561	2–1,065	99.79	1	4,965
PUfA	70	223,370	117,106	3–1,222	99.76	1	10,829

*Average coverage was calculated for every contig separately; # the *de novo* assembled draft genome of PBek isolate has coverage of ≥20

**Table 5:** Statistics of *de novo* assembled contigs with SPAdes *de novo* assembler.

Name	# denovo contigs (min length 500)	Max length	N50	Average coverage*	% of contigs with ≥10 coverage	# misassemblies	Total length of misassembled contigs
A17	25	707,517	707,517	5–813	99.64	3	77,978
PBek	32	932,242	932,242	1–1543	99.24^#^	2	52,829
PAbJ	41	639,612	639,612	1–848	99.59	1	4,580
PAbN	50	639,654	639,654	1–790	99.28	2	39,130
PMaC	42	639,628	639,628	1–915	99.61	1	4,866
PUfA	54	639,717	639,717	1–991	98.96	2	27,148

*Average coverage was calculated for every contig separately; # the *de novo* assembled draft genome of PBek isolate has coverage of ≥20

**Fig 3:**
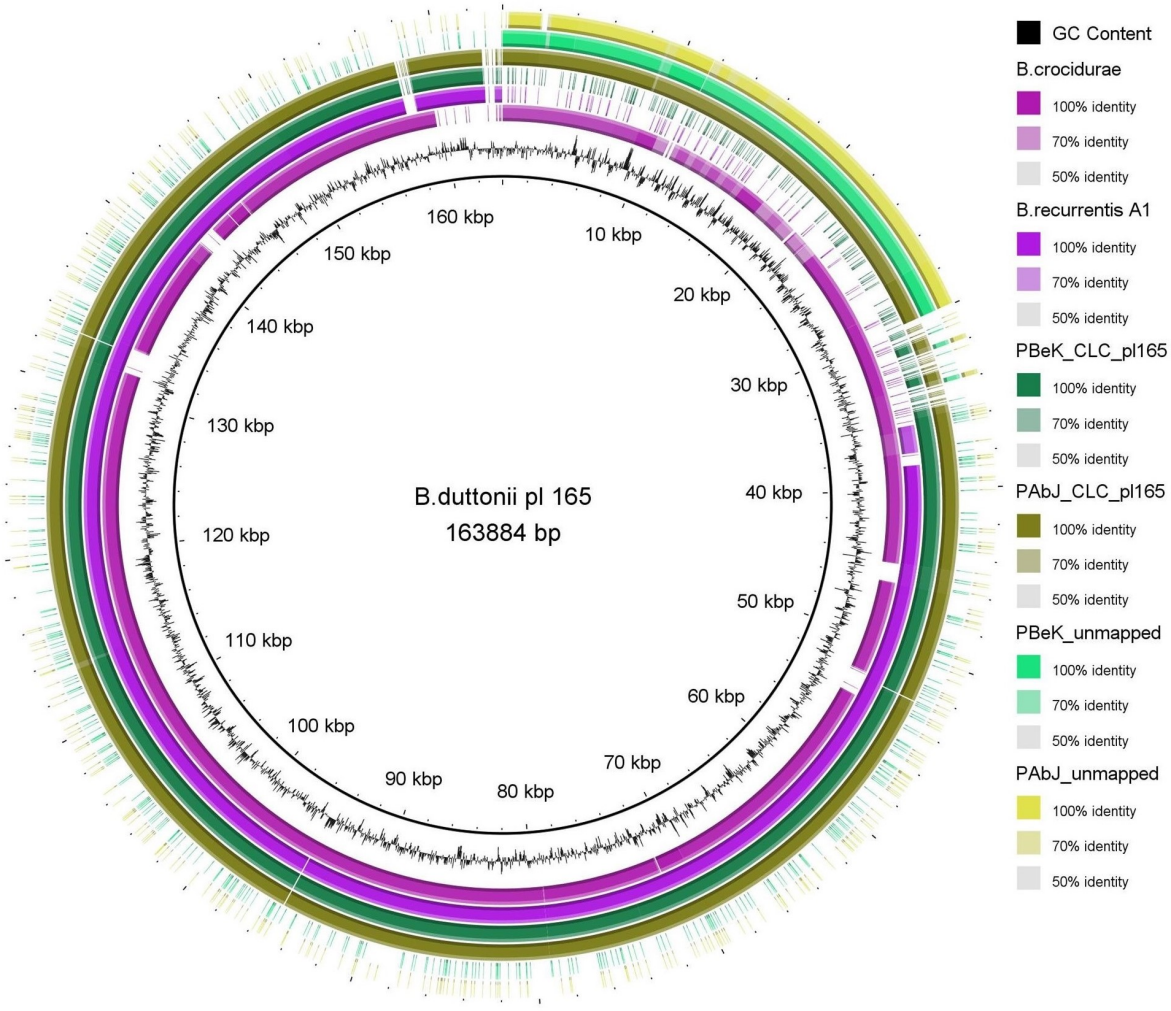
BRIG representation of the longest identified plasmid from *B*. *duttonii* pl165 in comparison to *B*. *crocidurae*, *B*. *recurrentis* A1 and the *de novo* assembled contigs assembled with the CLC assembler as exemplified by PBek, PAbJ and *de novo* assembled contigs from the 3% reads of the respective isolates not mapped to the reference strain A1 in the CLC mapping step.

Plasmid pl124 of *B*. *recurrentis* strain A1 was described as being co-linear to plasmid pl165 of *B*. *duttonii* strain Ly, but was 40 kb shorter [17]. A highly similar plasmid was detected also in a recently sequenced *B*. *crocidurae* [42] (Accession number: NC_017778.1). Evidence for a larger version of pl124 was present in all isolates examined in this study. Contigs assembled *de novo* from reads that did not map to the reference sequence (approx. 3%, Table 3) as well as *de novo* assembled contigs from raw reads matched the 40 kb on the left 5’ end of the pl165 from *B*. *duttonii* and *B*. *crocidurae* that was lacking in *B*. *recurrentis* strain A1 (Fig 3).

**Fig 4:**
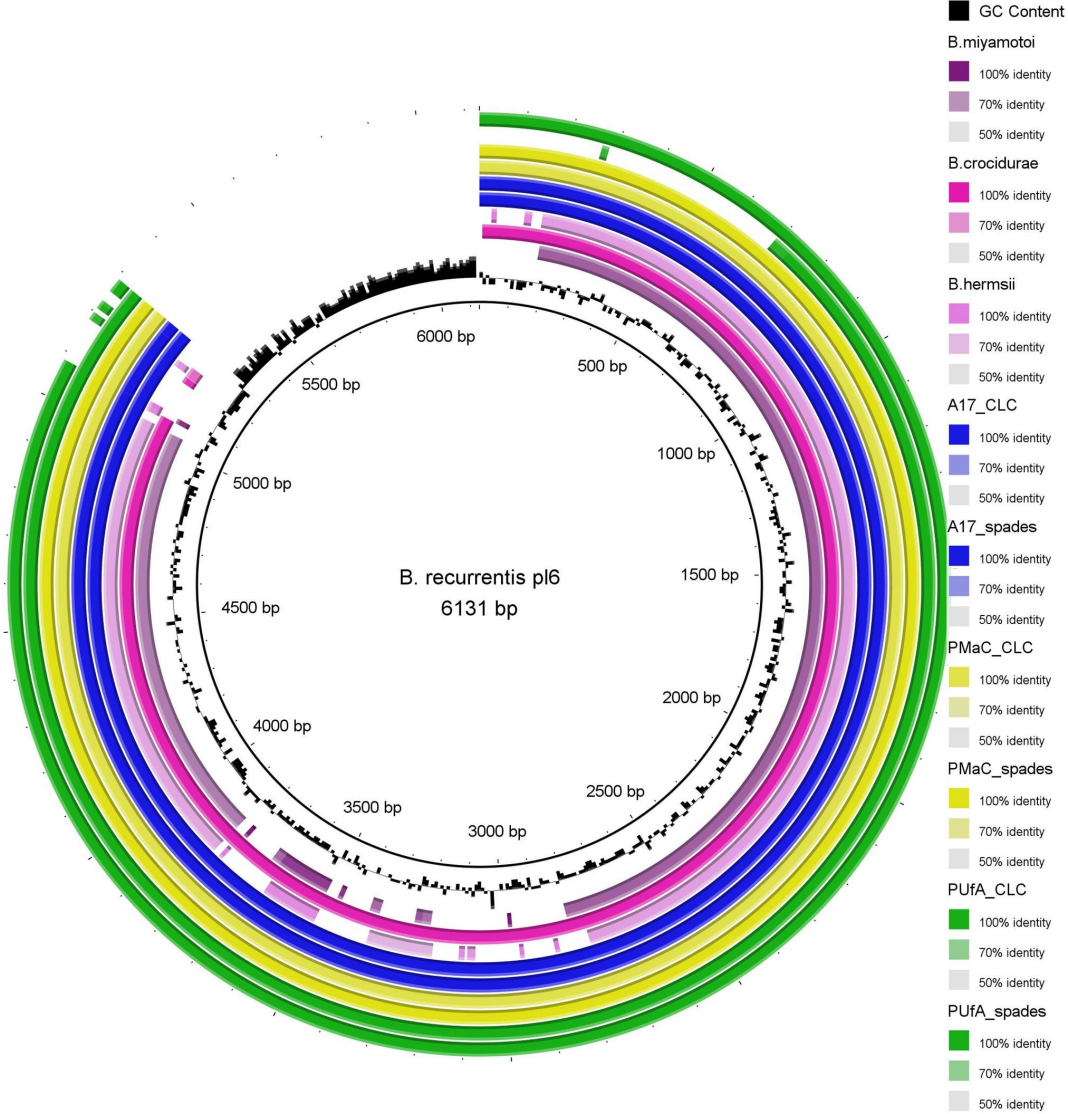
BRIG representation of the shortest identified plasmid from *B*. *recurrentis* A1 pl6 in comparison to *B*. *miyamotoi*, *B*. *crocidurae*, *B*. *hermsii* and the *de novo* assembled contigs assembled with the CLC or SPAdes assembler as exemplified with A17, PMaC and PUfa isolates.

The opposite was true for plasmid pl6. All sequenced isolates lacked approximately 1 kb at the 3´ end of the plasmid sequence (Fig 4.). The lacking sequence region contains two hypothetical proteins in the reference genome [17]. In order to rule out assembly errors, PCR primers were designed for the aforementioned regions in both plasmids. For pl6 no PCR products were obtained while for pl124/165, PCR products of the expected size were obtained in all samples (S1 Fig). These results confirmed the presence/absence of these regions in the plasmids of the *B*. *recurrentis* isolates investigated here. Furthermore, it was noticed that the reference genome of *B*. *duttonii* available in GenBank does not contain a counterpart of the smallest *B*. *recurrentis* plasmid, pl6, but that similar plasmids are present in the genomes of *B*. *crocidurae* [42](NC_017775.1), *B*. *miyamotoi* [43](KT355574.1) and *B*. *hermsii* (CP005739.1) (Fig 4).

## Discussion

Recent migrations to Europe brought new attention to a nearly forgotten disease—LBRF. The interest was raised both in clinicians and the research community. Cases of LBRF were reported in Germany [8], Italy [24], the Netherlands [25], Belgium [26], Finland [27] and Switzerland [28, 29]. All cases were imported, and associated with newly arrived migrants, with a possible autochtonuous transmission in Italy [24]. With a high incidence of LBRF in countries at the Horn of Africa, the disease is still a prominent challenge for the local public health systems. Due to several reasons such as the difficulties in isolating and *in vitro* culturing *B*. *recurrentis*, strict host specificity and lack of an animal model to properly study them *in vivo* [3], little is known about the genomic composition and intraspecific variability of *B*. *recurrentis*. Only one sequenced genome was publicly available so far for *B*. *recurrentis*, and typing methods are scarce and deduced from information available from closely related TBRF borreliae [15]. The number of cultured isolates available even now worldwide is very limited mostly due to the fact that they grow unsatisfactorily under *in vitro* conditions established for *Borrelia*. The increase of RF cases in Germany and Europe presented the opportunity to explore the genomic variability with state-of-the-art sequencing technologies and provide valuable information to further our understanding of this pathogen and to develop better diagnostic tools.

### Nucleotide variability on the chromosome of *B*. *recurrentis* is low

The variability at the SNP level found in our samples compared to the *B*. *recurrentis* A1 reference genome was low, and mostly located on the chromosome. High conservation was already observed among RF borreliae based on sequencing short segments of different genetic loci: 16S, *flaB*, *glpQ*, IGS [15], MST [44] and whole DNA-DNA hybridization studies [31]. The species *B*. *duttonii* and *B*. *recurrentis* are often indistinguishable by these methods with > 99% sequence identity, that was exemplified again by the low number of SNPs when mapping *B*. *recurrentis* reads to *B*. *duttonii* reference (n = 185–260). It has been hypothesized that they are in fact the same species with the latter having a shorter, decaying genome due to an inactive *recA* [17] involved in DNA doublestrand break repair as a result of the adaptation to new hosts (louse/human). Indeed, similar gene loss was also observed in other louse-borne bacterial species compared to their tick-borne counterpart, such as *Bartonella quintana* and *Bartonella henselae* [45], *Rickettsia prowazekii* and *Rickettsia conorii* [46]. Nevertheless, the identified SNPs could be further explored as possible targets for specific diagnostic and/or typing tools.

The identified 12–17 SNPs on the chromosome of the six sequenced isolates investigated here were not present in the 8 housekeeping genes used for the MLST typing of *Borrelia* species. Therefore, by using MLST typing all newly identified isolates would be indistinguishable from the *B*. *recurrentis* A1 reference strain, and would belong to the same MLST sequence type ST-669. This method still has the discriminatory power to distinguish *B*. *recurrentis* from *B*. *duttonii* and *B*. *crocidurae*. Employing other methods used to differentiate closely related *B*. *duttonii* and *B*. *recurrentis* would not provide more discriminatory power in the investigation of intraspecies variability in this study, as all isolates examined contained same sequences as the reference genome on the loci used for the MST and IGS, and therefore facing the same limitations as already noted for these methods [16, 40].

Interestingly, applying the IGS and MST typing methods to a higher number of samples from different sources did in fact find variability among *B*. *recurrentis*, separating them into two clades albeit by a single SNP difference [16, 40]. The isolates came from a limited number of cultured patient samples and DNA isolates obtained directly from lice [40] or DNA isolates obtained directly from patient blood samples [16]. Similar observations were made with the IGS method and *B*. *duttonii* isolates. In total, by means of IGS typing, four different clades could be identified among *B*. *duttonii* isolates from cultured isolates, DNA isolated directly from patient blood, or from ticks [40]. However, all cultured *B*. *duttonii* isolates belonged to the same clade implying that there might be a bias introduced with adaptation to culture, masking the real variability present in host and vector. A similar effect could have occurred for the six *B*. *recurrentis* isolates examined here. Although cultivable *B*. *recurrentis* isolates from two IGS subtypes were described in literature [40], all isolates sequenced in this study belong to only one IGS subtype and thus may not reflect the potential full extent of diversity. Furthermore, one might, speculate that the variability is higher amongst the bacterial population that is not cultivable from patient blood. The observed difficulties to adapt the pathogen to *in vitro* culture conditions would support this hypothesis: Out of 38 blood samples available in 2015 at the Bavarian Health and Food Safety Authority, 21 adapted to culture conditions. One needs to consider, that some patients were already treated with antibiotics prior to blood sampling and that this might have hampered recovery of *B*. *recurrentis* from blood. Only nine had grown to meaningful densities and four strains yielded sufficient amounts of DNA for sequencing. It is possible that culture adaptation acts as selective pressure and it may be that only specific variants/clones readily adapt to culture as observed for *B*. *duttonii* [40]. Therefore, the observed low variability in the currently investigated strains would be the variability of the variant/clone that grows readily under *in vitro* culture conditions. An alternative hypothesis to explain the low variability is that due to its adaptation to a new host and vector *B*. *recurrentis* underwent a strong population bottleneck that is still noticeable in the population today. There is no natural animal reservoir available to study the infection and virulence of this pathogen. In order to address these issues, it would be very interesting to collect lice from infected people and examine the variability of *B*. *recurrentis* in lice. Additionally, culture independent sequencing methods directly from patient blood and from vectors could be employed, to circumvent possible bias introduced by infection and/or culture [47].

The stringency of SNP calling in our study was high, in order to exclude SNPs resulting from sequencing error. Performing SNP calling with less stringency (minimum frequency 75% and 50%) identified two additional SNPs. At 75% a deletion in a poly-A region was detected in all isolates from 2015 and in the PBek historical isolate and at 50% in the PBek isolate one more SNP was called in a coding region. Both SNPs led to a frameshift and would hamper the expression of the given gene, therefore, we consider it highly likely that these SNPs were artefactual as they would render the resulting protein non-functional.

Out of the six SNPs that were common to all newly sequenced isolates compared to the reference genome, one was in the leading methionine AUG start codon of an UDP-N-acetylmuramoyl-tripeptide-D-alanyl-D-alanine ligase, changing it to a valine (GUG). However, this leading methionine codon was identified via automated gene prediction method, while the homologous genes in *B*. *duttonii* and *B*. *crocidurae* started with an alternative start codon UUG that is present three base pairs upstream of this hypothetical start codon. The sequence coding for the same alternative start codon was present also three base pairs upstream in the isolates examined here, therefore active transcription of this gene can be assumed in spite of the change in the alleged start codon identified via automated gene prediction method.

Even though the genomes of the different *B*. *recurrentis* isolates were highly conserved, the phylogenetic trees based on the SNPs (both only chromosomal, as well as total genomic SNPs) showed a clear distinction between the historical isolates and the isolates recovered in 2015 (Fig 1). The latter were all identical, bearing the same chromosomal SNPs in comparison to the reference strain and differing also from the two historical isolates A17 and PBek in 18 and 1 SNPs, respectively.

The epidemiological investigations pointed to a common infection source along the Mediterranean migration route for refugees from East Africa [8, 28]. The identical SNP pattern in refugee isolates supports this hypothesis, even in the light of the low variability of the *B*. *recurrentis* genome. Using the WGS approach and SNP analysis we were able to find a limited number of differences between the two isolates obtained at the same time and kept in culture (A1 and A17). These isolates were otherwise indistinguishable with other methods [15]. This underlines once more the value of WGS as a tool to examine phylogenetic relationships, especially when investigating pathogens with low genome variability.

### Plasmids in *B*. *recurrentis*

Compared to the seven linear plasmids of the A1 reference genome, the majority of SNPs identified through mapping were located at the ends of the plasmids and might have been introduced through sequencing errors either in the reference genome or the newly sequenced isolates. Interestingly, the highest number of SNPs (n = 10–14) was at the left 5’ end of plasmid pl53 in the coding sequence for a hypothetical protein (locus tag: BRE_RS05500). This hypothetical CDS showed high similarity to other genes coding for surface proteins (*vlp*, *vsp*, *vmp*) from relapsing fever *Borrelia*. The high SNP density at this particular site would support the already noted antigenic variation of these surface proteins, that occurs through gene conversion in the genomes of relapsing fever *Borrelia* in order to escape the host immune system[48, 49].

The proportion of reads that remained unmapped in our sequence analysis suggested that the reference genome deposited at GenBank, might not have been complete. *De novo* assemblies of both, unmapped reads and performed with all reads, revealed contigs similar to the longest plasmid, pl165, of TBRF *Borrelia* (*B*. *duttonii* and *B*. *crocidurae*). The assembled contigs matched to the left end of the plasmid, that was initially not identified in the *B*. *recurrentis* reference genome of strain A1. There are several possible explanations to this: (i) it may be the result of long adaptation to culture conditions, as already noted for *B*. *hermsii* and *B*. *turicatae* that spontaneously lost part of the largest plasmid after 100 serial passages *in vitro* without impeding their infectivity [50], (ii) it could also be attributed to sequencing and/or assembly error. We favour the last hypothesis for the following reasons: Initial studies performed on *B*. *recurrentis* cultivable isolates (A1-A18) and plasmid analysis with PFGE did suggest the presence of a large, approximately 180–190 kb long plasmid [20, 31]. In addition, the presence and functionality of a gene designated *cihC*, coding for an outer membrane lipoprotein that binds major inhibitors of the human complement activation system (C4b-binding protein and C1 esterase inhibitor) was shown to be present in both isolates (A1 and A17) and located on a ~200 kb long linear plasmid [51]. Orthologous genes are present on the left termini of the longest plasmids of *B*. *duttonii*, *B*. *hermsii* and *B*. *turicatae* genomes [51]. PCR with primers designed to overlap the border between pl124 and pl165 of *B*. *duttonii* resulted in PCR products of the expected size in all six here examined isolates. These data provide evidence in support of *B*. *recurrentis* possessing a longer plasmid of approximately 160 kbp than present in the currently available strain A1 in GenBank.

Although *B*. *recurrentis* is hypothesized to be a louse-adapted clone of *B*. *duttonii*, interestingly, there is no counterpart of the smallest plasmid pl6 in the *B*. *duttonii* genome. However, a highly similar plasmid is present in other tick-borne relapsing fever borreliae, i.e. *B*. *crocidurae*, *B*. *miyamotoi* and *B*. *hermsii*. To overcome the difficulties of distinguishing *B*. *duttonii* and *B*. *recurrentis* [15], it might be of interest to consider this plasmid as a potential target for diagnostic and/or typing tools. However, it would be necessary to investigate the stability and retention of this specific plasmid *in vivo* and *in vitro*. It is known that RF *Borrelia* retain infectivity and majority of their plasmids with minimal rearrangements on the largest plasmid even after 100 cycles of *in vitro* cultivation [50]. Furthermore, compared to the reference genome of strain A1, all of the currently examined isolates contained a shorter version of the plasmid of approximately 5 kb. The length of the *de novo* assembled contigs representative of this plasmid did show some differences depending on the assembly algorithm employed (as exemplified in Fig 4 with PUfA_CLC and PUfA_spades). However, irrespective of the assembly method employed (mapping, *de novo* assembly with CLC or SPAdes) the right 3’ end of the plasmid was not detected. PCR reactions with primers designed to this region were negative as well. We propose that it may have resulted from a contamination and/or sequencing error in the reference genome A1, as this region has a much higher GC % content compared to the rest of the plasmid (Fig 4).

### *De novo* assemblies of *B*. *recurrentis* isolates

Likely due to the low passage number the isolates investigated in this study contained in the raw reads a proportion of human DNA (30–60%; Table 3) which interfered with *de novo* assembly. Therefore all reads were first mapped to the human genome and the remaining unmapped reads were assembled using SPAdes or the CLC *de novo* assembler. This improved the assembly significantly and produced longer contigs, in some cases even covering the whole chromosome (PBek in Table 4). Although the chromosome was easily identified and assembled even with *de novo* assemblers, the assembly of plasmids was more tedious, similar as recently published for Lyme disease borrelia [52]. The contigs matching to the main chromosome were in synteny with the published reference genome (see supplementary data S1 Data). When comparing contigs representing plasmids from the two employed assemblers, it is unclear which performed better. CLC produced more and shorter contigs that aligned more unambiguously to the reference genome and had less identified misassemblies (Tables 4 and 5). On the other hand, SPAdes produced fewer and longer contigs which matched to regions in more than one plasmid of the reference A1 genome, thus rendering their location inconclusive (Tables 4 and 5). RF borreliae have a smaller number of plasmids than Lyme borreliosis group spirochetes [17]. However, RF borreliae contain several copies of the so called *vlp* and *vsp* genes present on different plasmids. *B*. *recurrentis* A1 strain has 17 intact *vlp* genes and 29 pseudogenes and 10 *vsp* genes [17]. These genes code for surface proteins that facilitate the evasion of the human immune system, and are responsible for the febrile relapses of the clinical picture of the disease [48, 49]. Due to both biological constraints and inherent methodological limitations of short-read sequencing and assembly, the construction of complete/whole plasmids in RF *Borrelia* could not have been unambiguously achieved with only *de novo* assembly approaches. The high sequence similarity and number of *vlp* and *vsp* genes present in the genome impeded the design of specific PCR primers necessary for the resolution of the position of ambiguous contigs matching to the location of two or more plasmids, as well as the size of the plasmids. Interestingly, when mapping the reads directly to the reference genome, a discrepancy in coverage of the regions of the genome containing *vlp* and *vsp* genes was observed. The coverage of the *vlp* and *vsp* genes was higher than the surrounding regions (10-20x higher), which would be suggestive of the presence of even more copies of the *vlp* and *vsp* genes, than previously reported in the reference genome, similar as already observed for the differing size of plasmid pl124 [51].

New and powerful emerging technologies that exploit long-read sequencing and hybrid assemblers combining long and short reads could offer more insights and allow the elaboration of the exact number and location of *vlp* and *vsp* genes, as well as the length of the plasmids in RF borreliae, as already done in a proof-of-concept manner for *Borrelia burgdorferi* [52].

Nevertheless, the currently obtained sequences are a valuable contribution in the field of RF *Borrelia* research and could present the basis for the development of molecular tools to facilitate the diagnostics and characterization of LBRF *Borrelia*.

## Supporting information

S1 Table Position of all SNPs compared to the reference genome *B*. *recurrentis* A1. (DOCX)

S1 Data MUMmer plots of reference *B*. *recurrentis* A1 and the *de novo* assemblies of the 6 sequenced isolates assembled with CLC or SPAdes. (ZIP)

S1 Fig Electrophoresis gel of amplified PCR products with two distinct primer sets for the detection of plasmid pl124 and pl6. (JPG)
