# Supplementary material for: First insights in the variability of Borrelia recurrentis genomes
Source: PLoS Negl Trop Dis. 2017 Sep 13;11(9):e0005865. doi: 10.1371/journal.pntd.0005865 (PMC5612729; doi:10.1371/journal.pntd.0005865)
Supplement: S1 Table — (DOCX) [file pntd.0005865.s001.docx]

Supplementary Table 1. Position of all SNPs compared to the reference genome *B. recurrentis* A1

| Strains containing the SNP | Reference Accession Number | Producttic element | Reference Position | Type | Ref | Allele | Overlapping annotations | Coding region change | Amino acid change |
| --- | --- | --- | --- | --- | --- | --- | --- | --- | --- |
| A17 | NC_011244 | chromosome | 114011 | SNV | C | T | Gene: BRE_RS00560, Product: serine protease | WP_041178076.1:c.846G>A | WP_041178076.1:p.Met282Ile |
| All | NC_011244 | chromosome | 114019 | SNV | A | C | Gene: BRE_RS00560, Product: serine protease | WP_041178076.1:c.838T>G | WP_041178076.1:p.Ser280Ala |
| PBek, PAbJ, PAbN, PUfA, PMaC | NC_011244 | chromosome | 142599 | SNV | T | C | Gene: BRE_RS00700, Product: transcript cleavage factor | WP_012538682.1:c.445A>G | WP_012538682.1:p.Asn149Asp |
| PAbJ, PAbN, PUfA, PMaC, | NC_011244 | chromosome | 151956 | SNV | C | T | Gene: BRE_RS00735, Product: acriflavin resistance protein | WP_012538687.1:c.2482G>A | WP_012538687.1:p.Val828Ile |
| A17 | NC_011244 | chromosome | 238406 | SNV | C | T | Gene: BRE_RS01125, Product: FliG | WP_041178095.1:c.1165G>A | WP_041178095.1:p.Val389Ile |
| A17 | NC_011244 | chromosome | 245969 | SNV | C | T | Gene: BRE_RS01155, Product: peptidase M16 | WP_012538752.1:c.1784C>T | WP_012538752.1:p.Thr595Ile |
| All | NC_011244 | chromosome | 336041 | SNV | T | C | Gene: BRE_RS01580, Product: UDP-N-acetylmuramoylalanyl-D-glutamyl-2, 6-diaminopimelate-D-alanyl-D-alanine ligase | WP_041178108.1:c.1A>G | WP_041178108.1:p.Met1? |
| PBek, PAbJ, PAbN, PUfA, PMaC | NC_011244 | chromosome | 365244 | SNV | G | A | Gene: BRE_RS01715, Product: dipeptide/oligopeptide/nickel ABC transporter ATP-binding protein | WP_012538826.1:c.92G>A | WP_012538826.1:p.Arg31His |
| A17 | NC_011244 | chromosome | 456488 | SNV | C | A | Gene: BRE_RS02120, rRNA: rRNA |  |  |
| PBek, PAbJ, PAbN, PUfA, PMaC | NC_011244 | chromosome | 474294 | SNV | G | A | Gene: gyrB, Product: gyrB | WP_041178119.1:c.443C>T | WP_041178119.1:p.Thr148Met |
| PBek, PAbN, PUfA, PMaC | NC_011244 | chromosome | 496870 | SNV | G | A | Gene: uvrC, Product: uvrC | WP_012538917.1:c.232C>T |  |
| A17 | NC_011244 | chromosome | 517980 | SNV | G | A | non-coding region |  |  |
| PBek, PAbJ, PAbN, PUfA, PMaC | NC_011244 | chromosome | 524522 | SNV | G | A | Gene: BRE_RS02525, Product: 30S ribosomal protein S5 | WP_025400767.1:c.469G>A | WP_025400767.1:p.Gly157Ser |
| PBek, PAbJ, PAbN, PUfA, PMaC | NC_011244 | chromosome | 600416 | SNV | G | T | Gene: BRE_RS02870, Product: chemotaxis protein CheW | WP_012538995.1:c.180G>T |  |
| PBek, PAbJ, PAbN, PUfA, PMaC | NC_011244 | chromosome | 655967 | SNV | A | G | Gene: BRE_RS03100, Product: trigger factor | WP_012539032.1:c.21A>G |  |
| PBek, PAbJ, PAbN, PUfA, PMaC | NC_011244 | chromosome | 739037 | SNV | C | T | Gene: BRE_RS03450, Product: chemotaxis protein | WP_012539087.1:c.2044C>T | WP_012539087.1:p.Pro682Ser |
| PBek, PAbJ, PAbN, PUfA, PMaC | NC_011244 | chromosome | 756395 | SNV | C | T | Gene: BRE_RS03540, Product: tRNA methyltransferase TrmD | WP_012538438.1:c.427C>T | WP_012538438.1:p.Leu143Phe |
| A17 | NC_011244 | chromosome | 812450 | SNV | G | T | Gene: BRE_RS03795, Product: ABC transporter permease | WP_012539133.1:c.704C>A | WP_012539133.1:p.Ser235Tyr |
| All | NC_011244 | chromosome | 813667 | SNV | A | C | non-coding region |  |  |
| PBek, PAbJ, PAbN, PUfA, PMaC | NC_011244 | chromosome | 867269 | SNV | G | A | Gene: BRE_RS04080, Product: transcription termination/antitermination protein NusA | WP_041178169.1:c.199G>A | WP_041178169.1:p.Glu67Lys |
| All | NC_011244 | chromosome | 930867 | SNV | C | T | Gene: BRE_RS04310, Product: NCS2 family permease | WP_012539217.1:c.654C>T |  |
| All | NC_011244 | chromosome | 930947 | SNV | T | G | Gene: BRE_RS04310, Product: NCS2 family permease | WP_012539217.1:c.734T>G | WP_012539217.1:p.Ile245Ser |
| All | NC_011244 | chromosome | 930964 | SNV | G | A | non-coding region |  |  |
| PAbJ, PAbN, PUfA, PMaC | NC_011246 | pl124 | 94331 | SNV | G | A | Gene: BRE_RS04770, Product: holin, BlyB family | WP_012539329.1:c.345C>T |  |
| PBek, PAbN, PUfA, PMaC | NC_011246 | pl124 | 107538 | Del | AT | - | Gene: BRE_RS04830, Product: hypothetical protein | BRE_RS04830:c.1313_1314delAT | BRE_RS04830:p.Tyr440fs |
| PBek, PAbJ, PAbN, PUfA, PMaC | NC_011246 | pl124 | 112147 | SNV | C | T | Gene: BRE_RS04845, Product: hypothetical protein | WP_012539337.1:c.1085C>T | WP_012539337.1:p.Ala362Val |
| PBek, PAbJ, PAbN, PUfA, PMaC | NC_011252 | pl23 | 7765 | SNV | A | G | Product: BRE_RS04925, Gene: hypothetical protein | BRE_RS04925:c.151A>G | BRE_RS04925:p.Lys51Glu |
| PAbJ, PAbN, PUfA, PMaC | NC_011252 | pl23 | 15792 | SNV | C | A | non-coding region |  |  |
| PBek, PUfA | NC_011252 | pl23 | 22925 | SNV | A | C | non-coding region |  |  |
| All | NC_011253 | pl33 | 11 | SNV | C | A | non-coding region |  |  |
| All | NC_011253 | pl33 | 56 | SNV | T | A | non-coding region |  |  |
| A17, PBek, PAbJ | NC_011255 | pl37 | 2082 | SNV | G | A | Repeat region |  |  |
| A17, PBek, PMaC | NC_011260 | pl53 | 165 | SNV | A | T | non-coding region |  |  |
| A17, PBek, PAbN, PUfA, PMaC | NC_011260 | pl53 | 170 | SNV | G | T | non-coding region |  |  |
| A17, PBek, PAbJ, PUfA, PMaC | NC_011260 | pl53 | 196 | Inser | - | A | non-coding region |  |  |
| A17, PBek, PAbJ, PUfA, PMaC | NC_011260 | pl53 | 197 | SNV | G | C | non-coding region |  |  |
| A17, PBek, PAbJ, PUfA, PMaC | NC_011260 | pl53 | 199 | Del | G | - | non-coding region |  |  |
| All | NC_011260 | pl53 | 202 | SNV | G | C | non-coding region |  |  |
| All | NC_011260 | pl53 | 208 | SNV | G | A | Gene: BRE_RS05500, Product: hypothetical protein | WP_041178256.1:c.206C>T | WP_041178256.1:p.Ala69Val |
| All | NC_011260 | pl53 | 216 | SNV | G | A | Gene: BRE_RS05500, Product: hypothetical protein | WP_041178256.1:c.201C>T |  |
| All | NC_011260 | pl53 | 221 | SNV | G | A | Gene: BRE_RS05500, Product: hypothetical protein | WP_041178256.1:c.198_199delACinsTA | WP_041178256.1:p.Lys66_Leu67delinsAsnIle |
| All | NC_011260 | pl53 | 223 | MNV | GT | TA | Gene: BRE_RS05500, Product: hypothetical protein | WP_041178256.1:c.189C>T |  |
| All | NC_011260 | pl53 | 233 | SNV | G | A | Gene: BRE_RS05500, Product: hypothetical protein | WP_041178256.1:c.182C>G | WP_041178256.1:p.Pro61Arg |
| All | NC_011260 | pl53 | 240 | SNV | G | C | Gene: BRE_RS05500, Product: hypothetical protein | WP_041178256.1:c.158delC | WP_041178256.1:p.Ser53fs |
| All | NC_011260 | pl53 | 264 | Del | G | - | non-coding region |  |  |
| All | NC_011260 | pl53 | 52739 | SNV | A | T | non-coding region |  |  |
