# Supplementary figures and images for: First insights in the variability of Borrelia recurrentis genomes
Source: PLoS Negl Trop Dis. 2017 Sep 13;11(9):e0005865. doi: 10.1371/journal.pntd.0005865 (PMC5612729; doi:10.1371/journal.pntd.0005865)

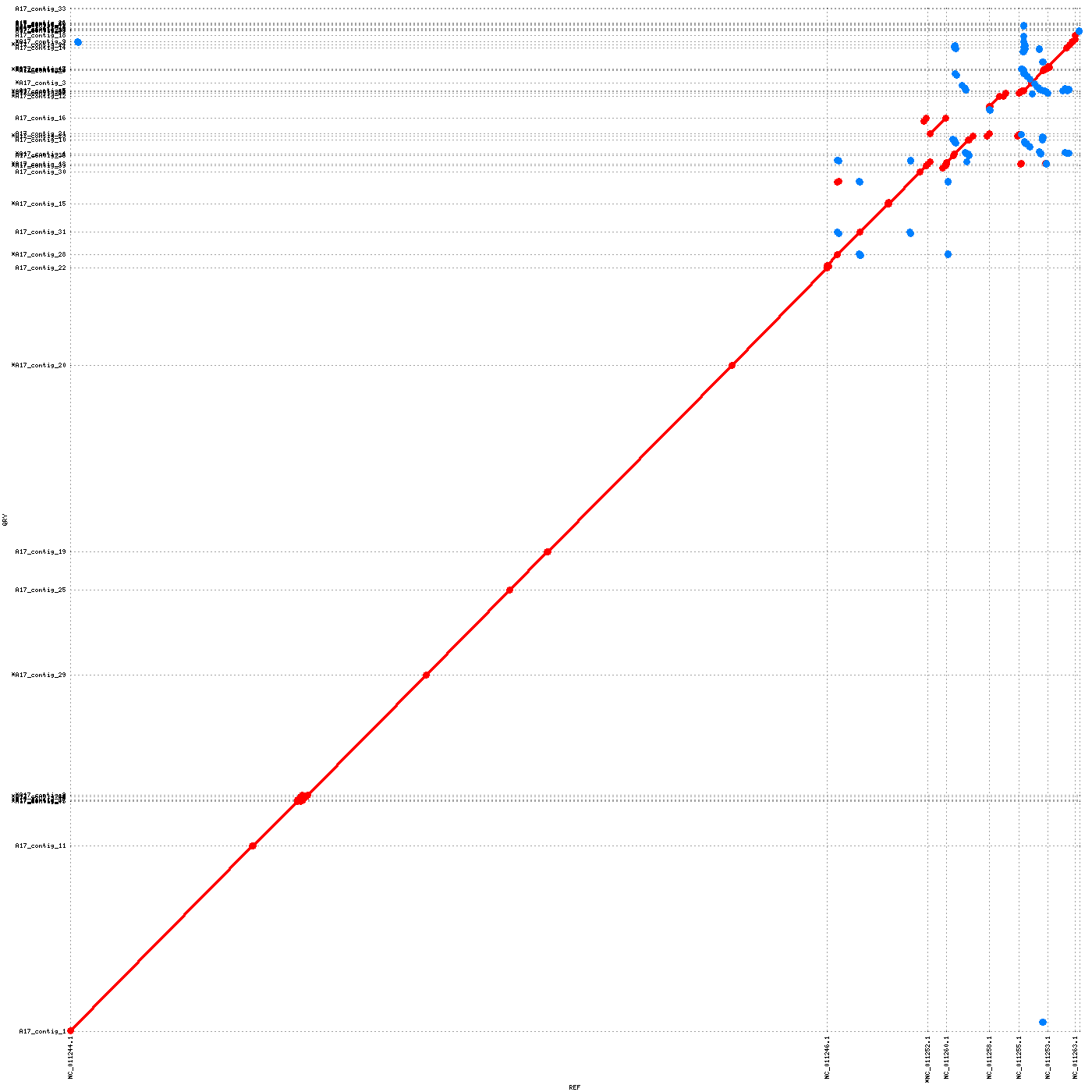

Supplement: S1 Data — (ZIP) [file pntd.0005865.s002.zip › out_A1_A17_CLC.png]

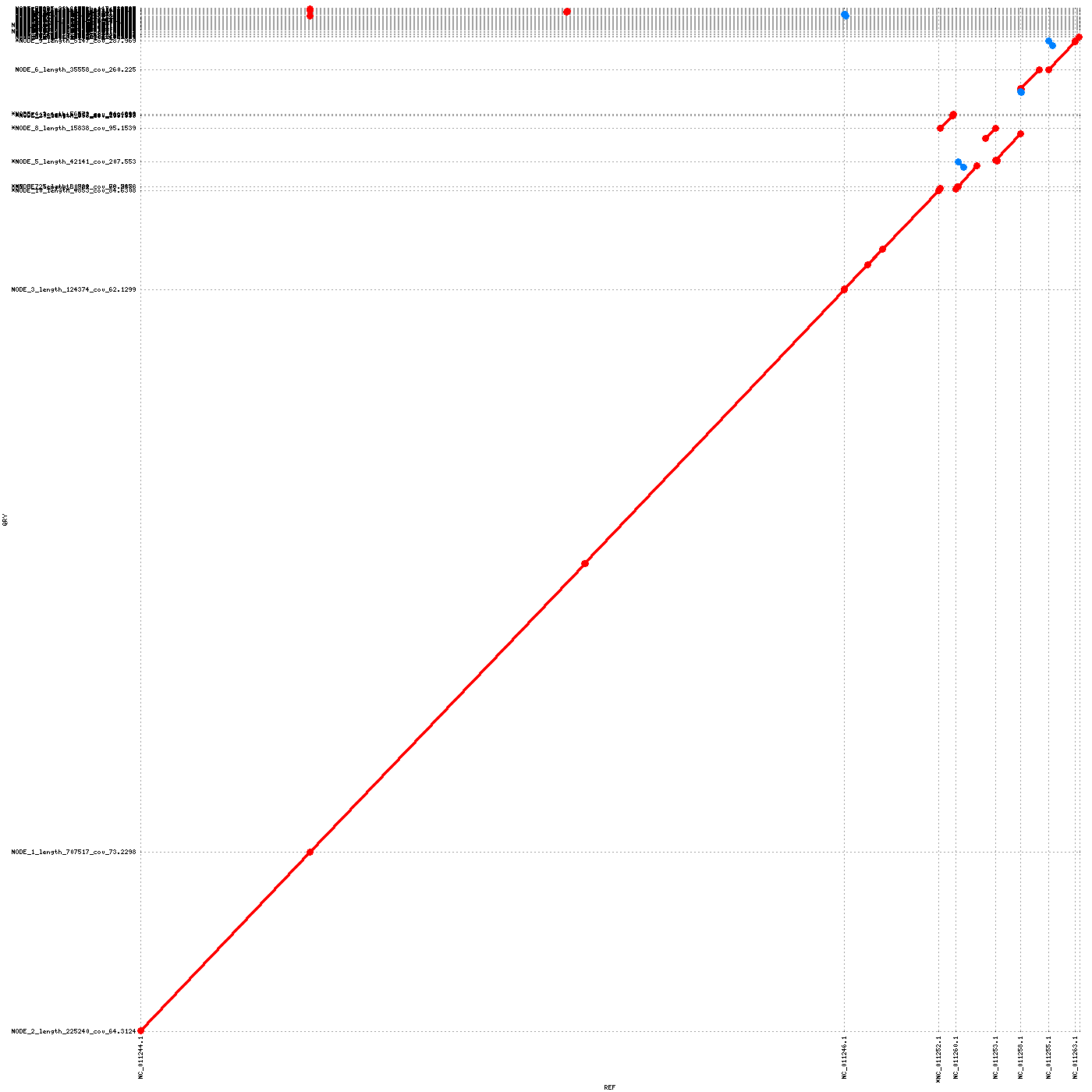

Supplement: S1 Data — (ZIP) [file pntd.0005865.s002.zip › out_A1_A17_spades.png]

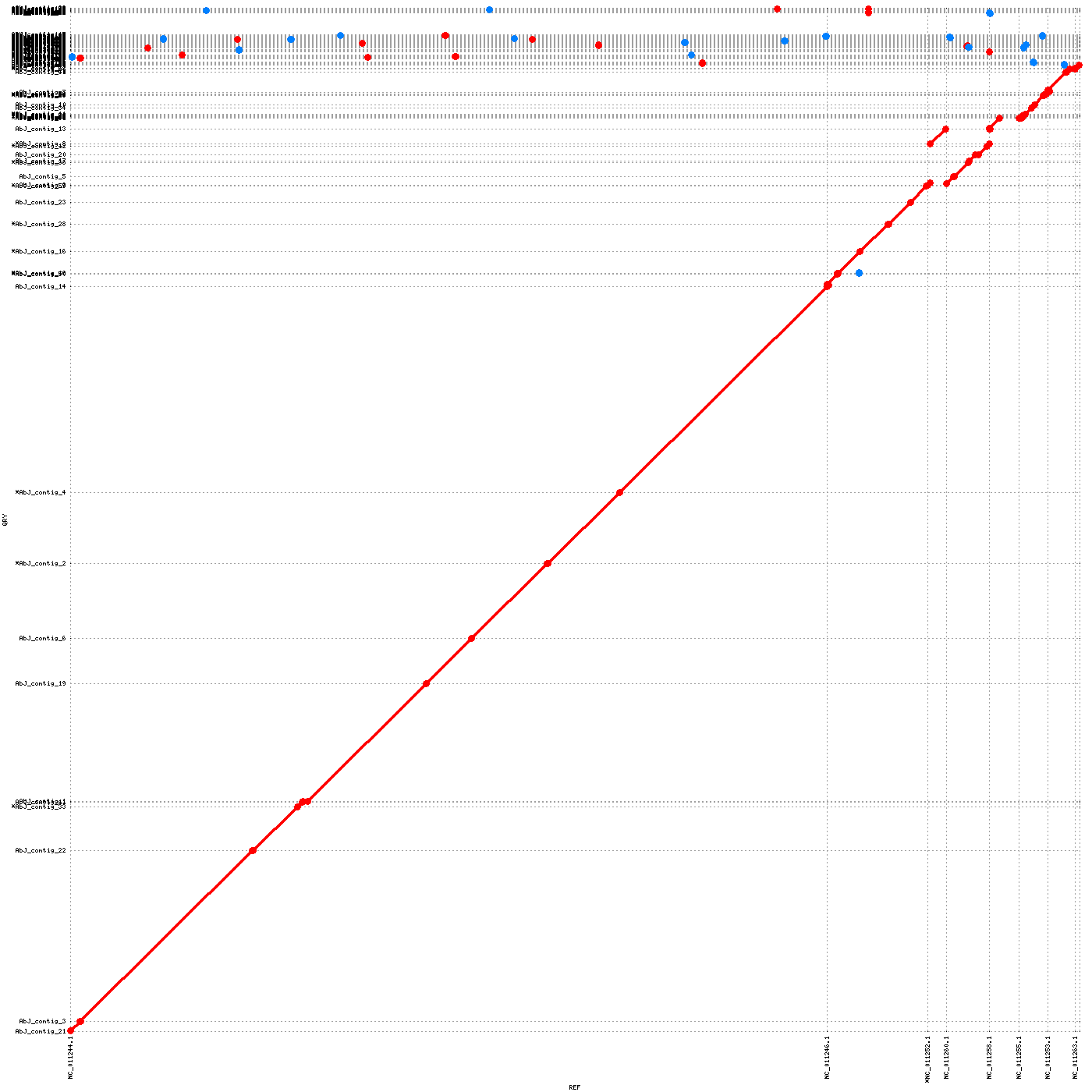

Supplement: S1 Data — (ZIP) [file pntd.0005865.s002.zip › out_A1_AbJ_CLC.png]

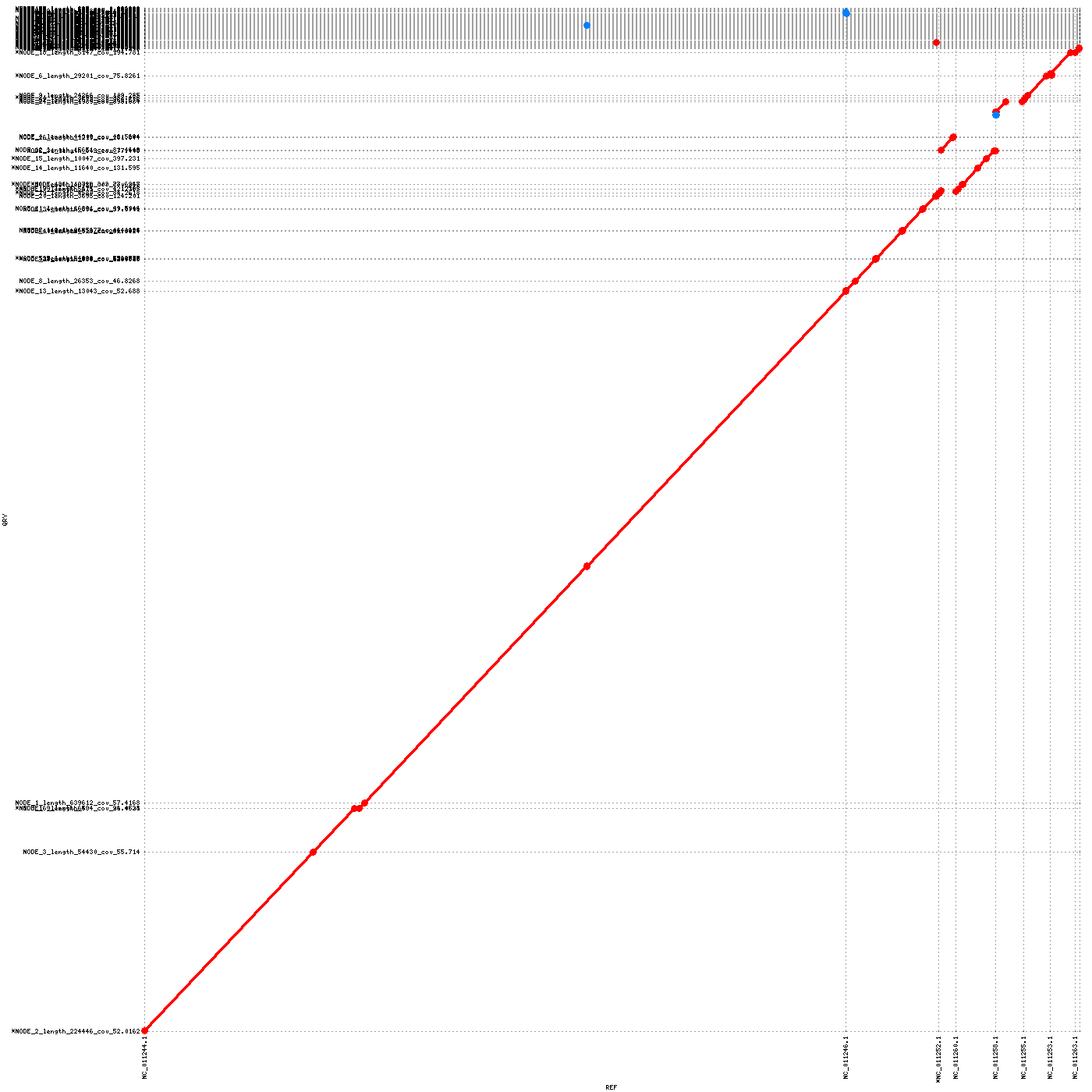

Supplement: S1 Data — (ZIP) [file pntd.0005865.s002.zip › out_A1_AbJ_spades.png]

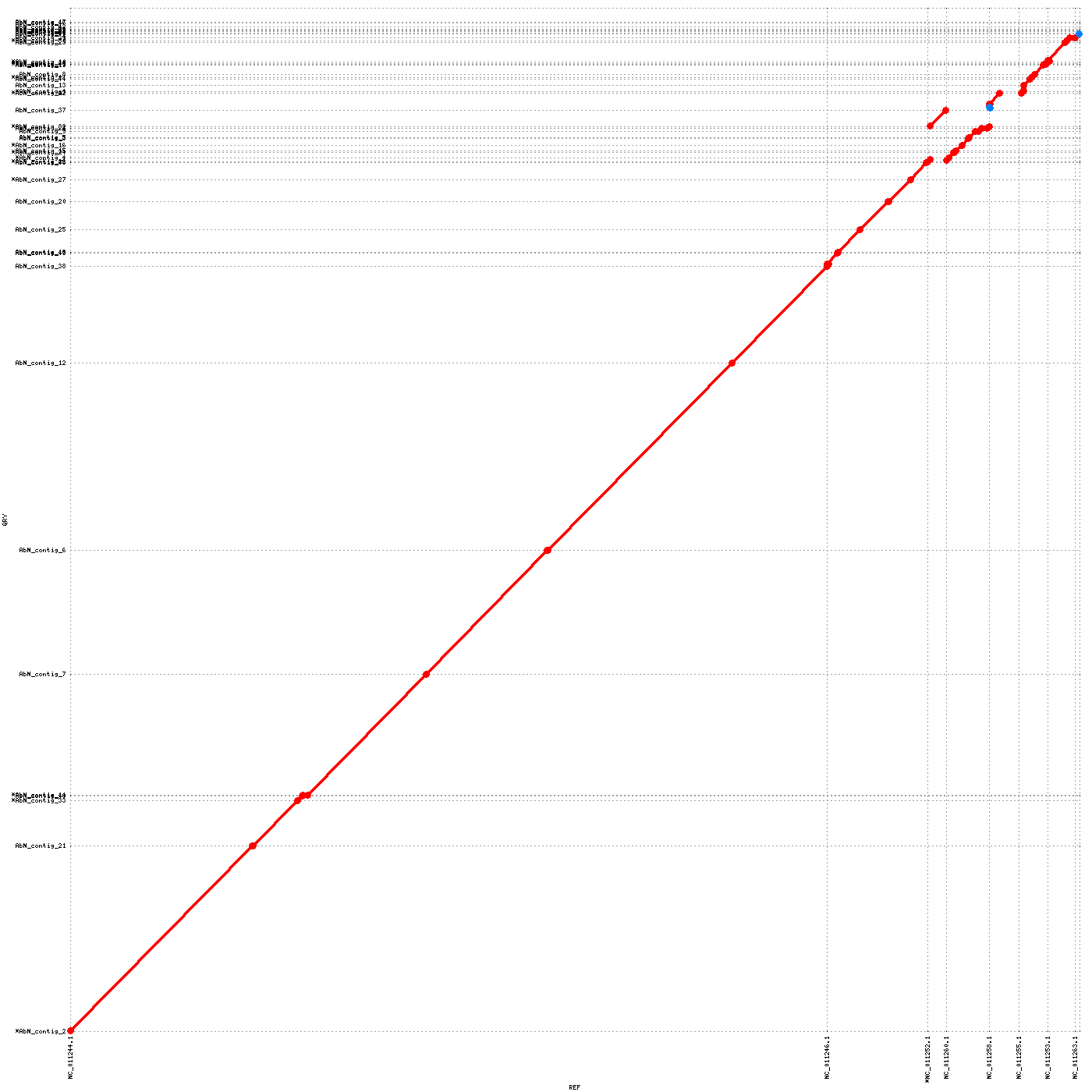

Supplement: S1 Data — (ZIP) [file pntd.0005865.s002.zip › out_A1_AbN_CLC.png]

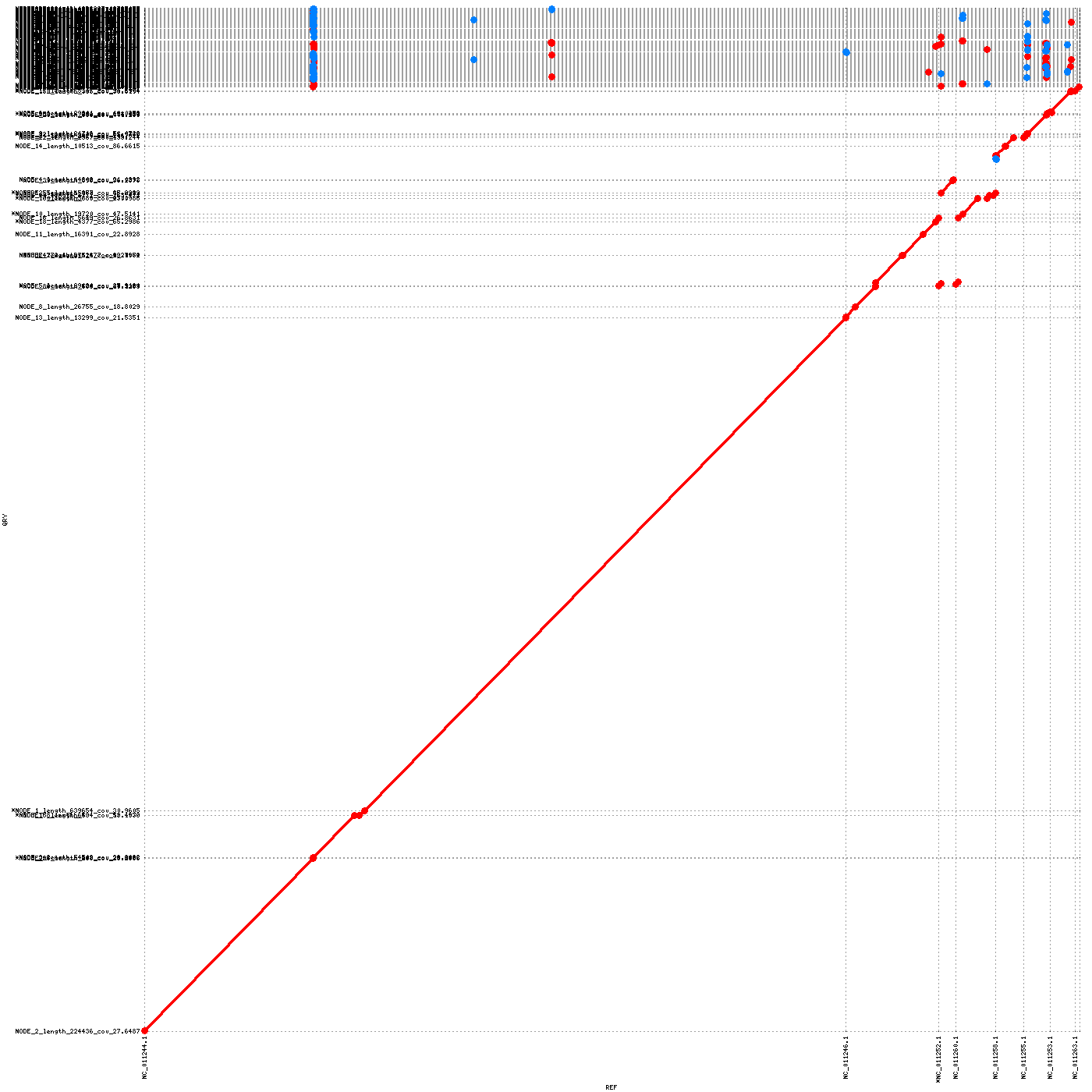

Supplement: S1 Data — (ZIP) [file pntd.0005865.s002.zip › out_A1_AbN_spades.png]

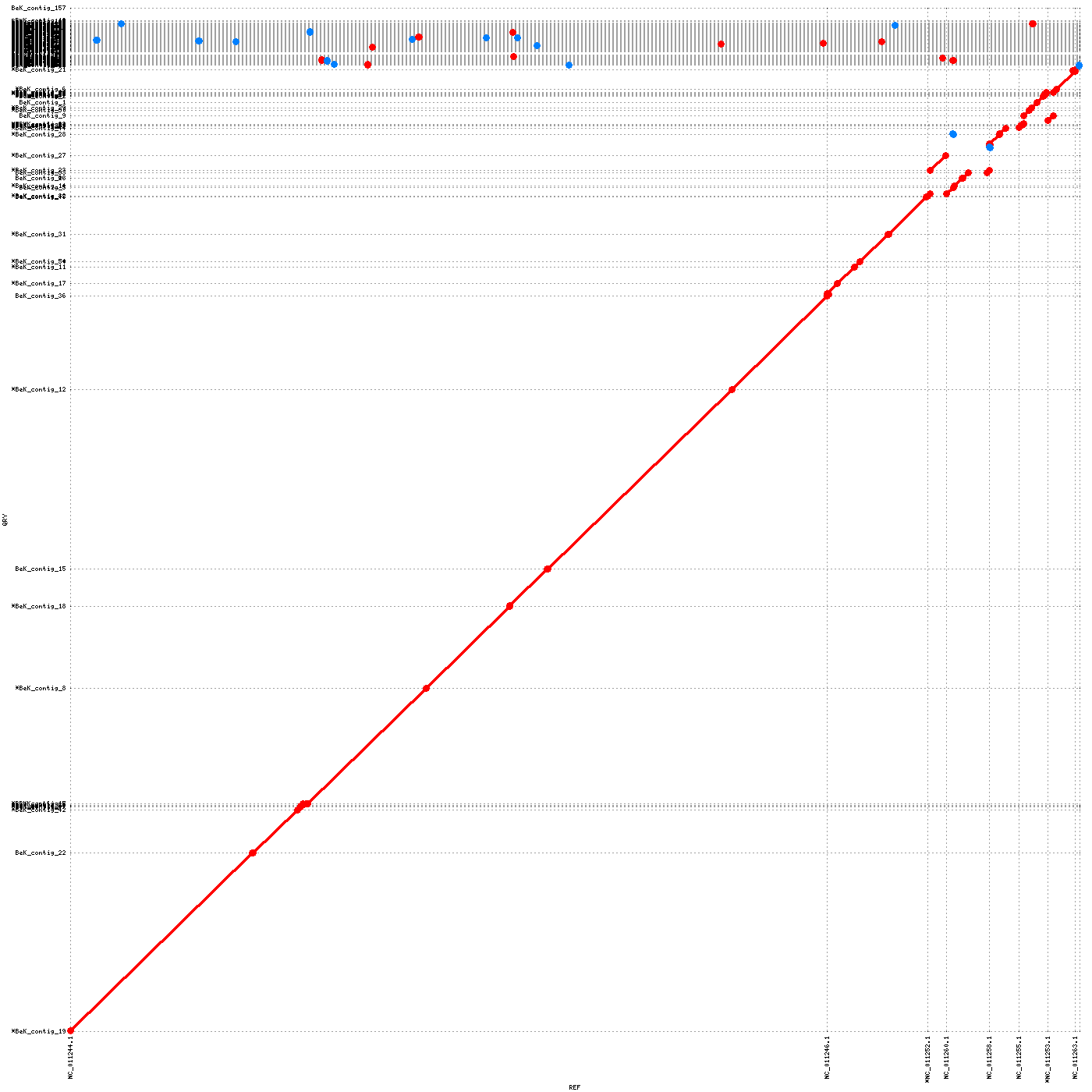

Supplement: S1 Data — (ZIP) [file pntd.0005865.s002.zip › out_A1_BeK_CLC.png]

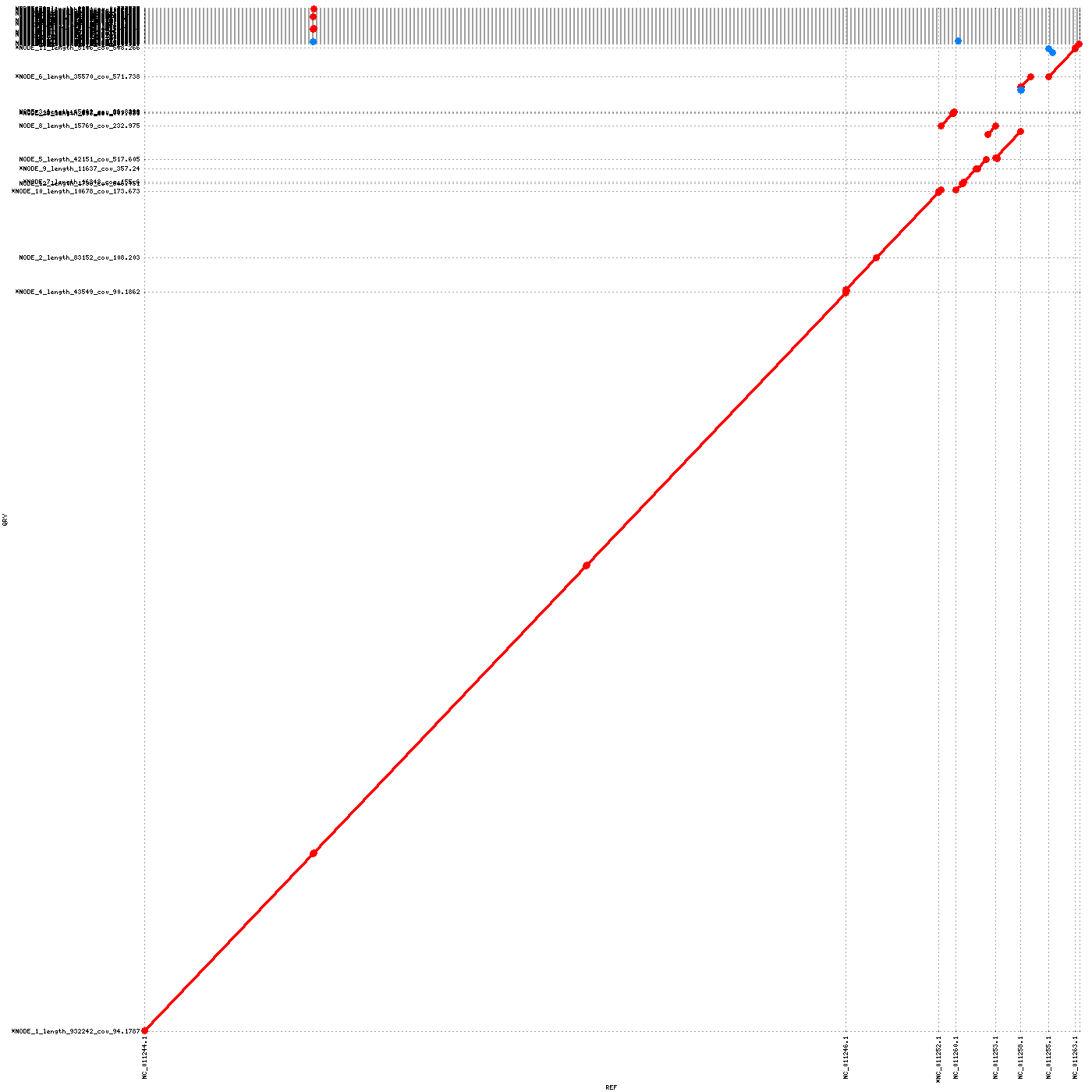

Supplement: S1 Data — (ZIP) [file pntd.0005865.s002.zip › out_A1_BeK_spades.png]

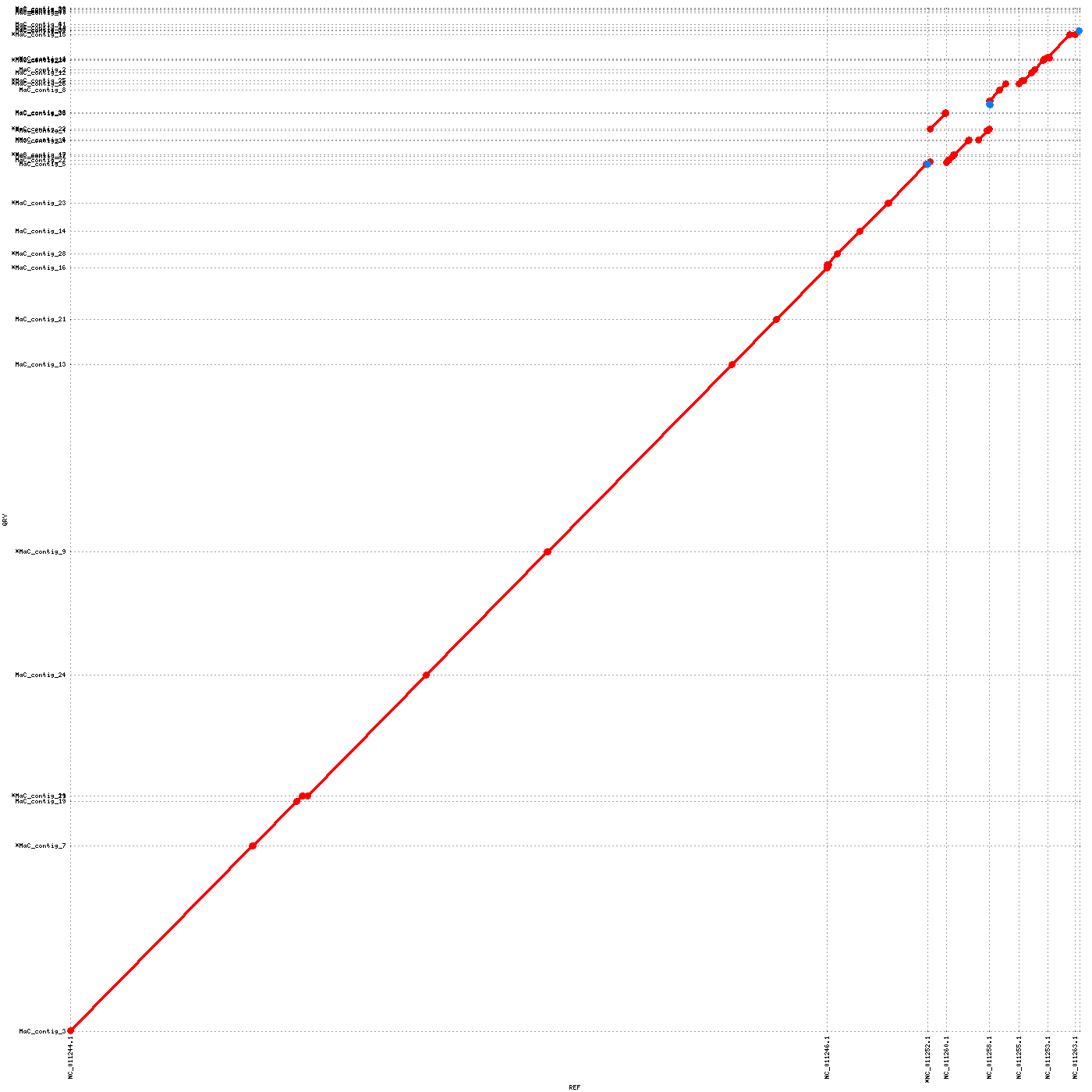

Supplement: S1 Data — (ZIP) [file pntd.0005865.s002.zip › out_A1_MaC_CLC.png]

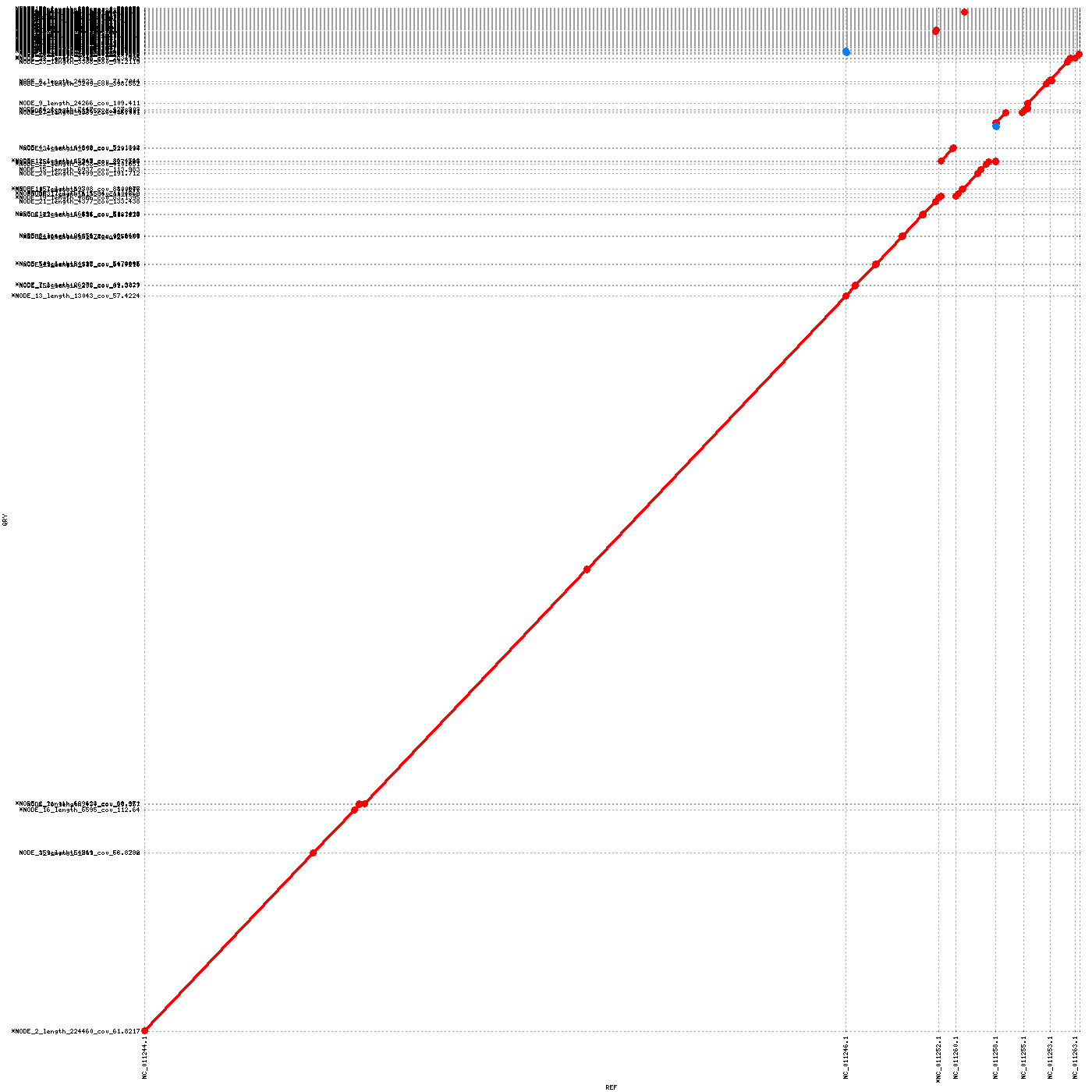

Supplement: S1 Data — (ZIP) [file pntd.0005865.s002.zip › out_A1_MaC_spades.png]

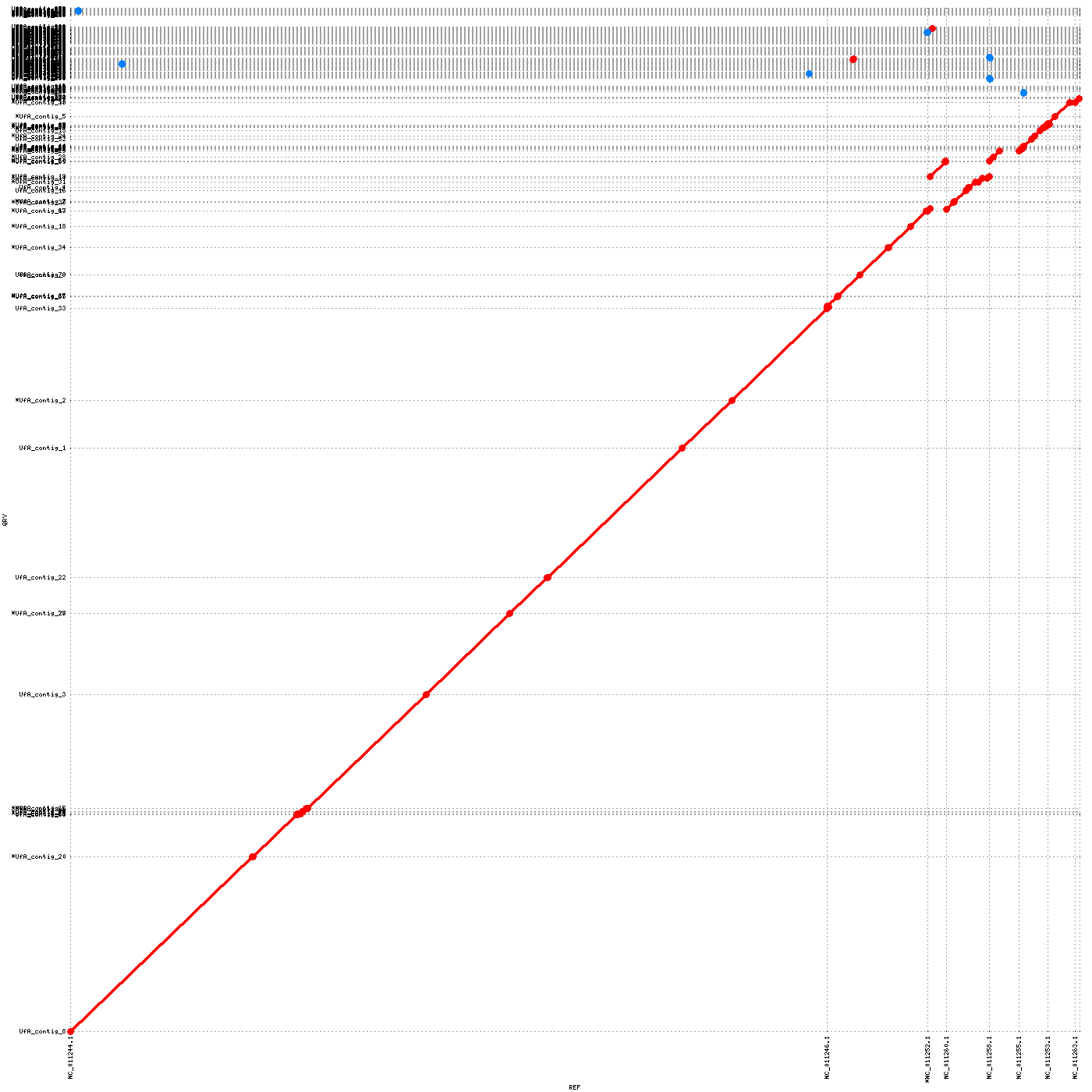

Supplement: S1 Data — (ZIP) [file pntd.0005865.s002.zip › out_A1_UfA_CLC.png]

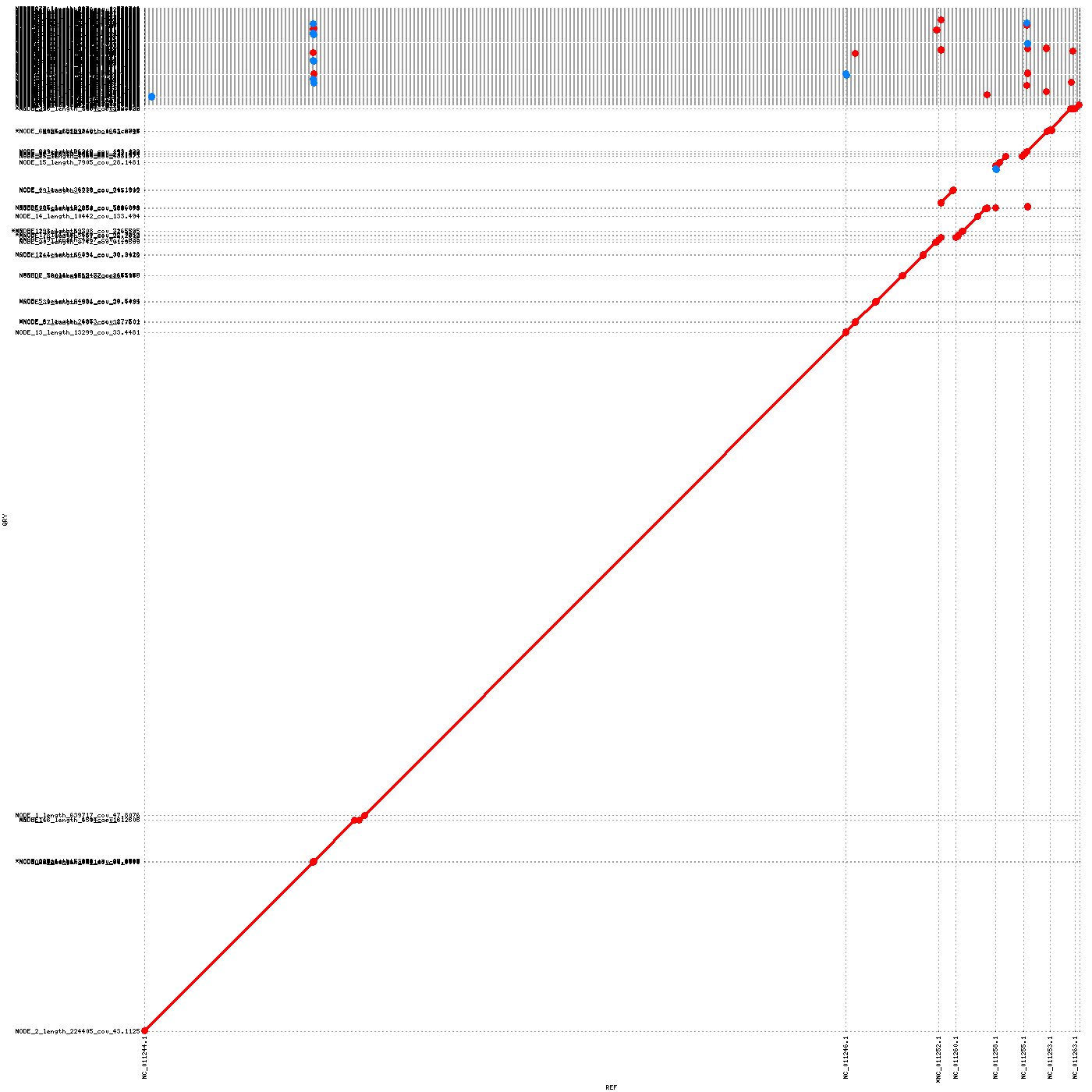

Supplement: S1 Data — (ZIP) [file pntd.0005865.s002.zip › out_A1_UfA_spades.png]

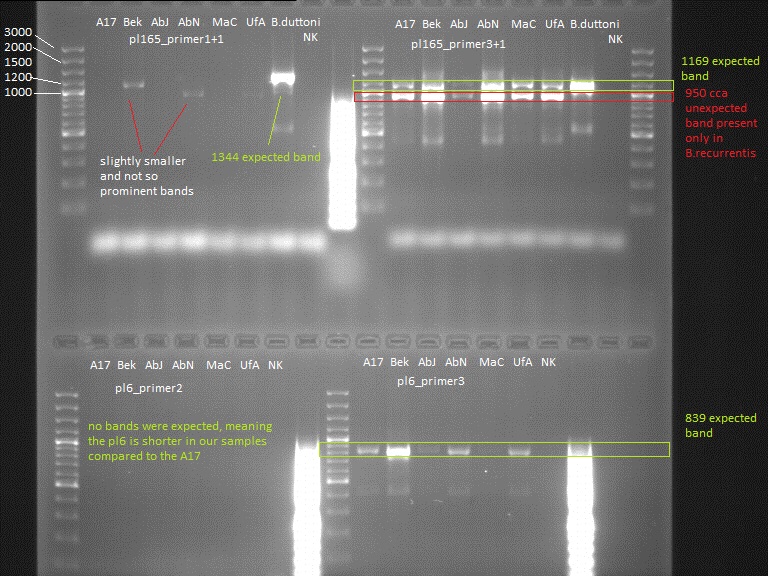

Supplement: S1 Fig — (JPG) [file pntd.0005865.s003.jpg]
